# Sexual and gender minority health in Chile: findings from the 2016–2017 Health Survey

**DOI:** 10.11606/s1518-8787.2022056004086

**Published:** 2022-11-18

**Authors:** Lilian Ferrer Lagunas, Billy Caceres, Margarita Bernales Silva, Alvaro Passi-Solar, Jaime Barrientos Delgado, Francisca López-Salvo, Ruby Shah, Tonda L. Hughes

**Affiliations:** I Pontificia Universidad Católica de Chile Facultad de Medicina Escuela de Enfermería Santiago Chile Pontificia Universidad Católica de Chile. Facultad de Medicina. Escuela de Enfermería. Santiago, Chile; II Columbia University School of Nursing Sexual and Gender Minority Research Center New York United States Columbia University School of Nursing. Sexual and Gender Minority Research Center. New York, United States; III University College London Research Department of Epidemiology and Public Health London United Kingdom University College London. Research Department of Epidemiology and Public Health. London, United Kingdom; IV Pontificia Universidad Católica de Chile Facultad de Medicina Departamento de Salud Pública Santiago Chile Pontificia Universidad Católica de Chile. Facultad de Medicina. Departamento de Salud Pública. Santiago, Chile; V Universidad Alberto Hurtado Facultad de Psicología Santiago Chile Universidad Alberto Hurtado. Facultad de Psicología. Santiago, Chile

**Keywords:** Sexual and Gender Minorities, Gender and Health, Risk Factors, Health Surveys

## Abstract

**OBJECTIVE:**

To expose visibility of the health concerns of sexual and gender minority groups in Chile, as well as to provide a platform to advocate for policies that support the health and well-being of sexual and gender minority people in the country.

**METHODS:**

The health conditions and risk factors of participants identified as sexual and gender minority were compared to those identified as cisgender heterosexual using data from the 2016-2017 National Health Survey.

**RESULTS:**

Despite reporting higher self-rated health than heterosexual men, gay men had a higher risk of lifetime diagnosis of sexually transmitted infections. Compared to heterosexual women, the prevalence of depression was higher among bisexual women, who were also less likely to have been tested for HIV. Moreover, transgender participants were more likely to report depression and worse self-rated health than cisgender heterosexual participants.

**CONCLUSION:**

Small sample sizes of sexual and gender minority subgroups might have obscured some differences that would have been observable in larger samples. Despite this, we found statistically significant sexual and/or gender identity differences in several health areas, especially mental, sexual, and overall health.

## INTRODUCTION

Over the past 10 years, global health organizations^1^ have begun to promote the health of sexual (e.g., lesbian, gay, and bisexual people) and gender (e.g., transgender and non-binary) minorities in an effort to reduce health disparities – that is, differences in health and healthcare provided for different population groups. Disparities vary based on a number of demographic factors, including race/ethnicity, socioeconomic status, age, sex, sexual orientation and gender identity.

The term LGBT – which refers to lesbian, gay, bisexual, and transgender people – has been used almost exclusively as an umbrella term to describe the larger population of sexual and gender minority (SGM) people. The newer term, SGM, includes LGBT people as well as those who do not identify as heterosexual, lesbian, gay, or bisexual; those whose gender identity differs from their assigned sex at birth; and those who reject traditional conceptualizations of gender in terms of the male-female dichotomy, thus being increasingly used, including by the United States (US) National Institutes of Health. Thus, we adopted the use of SGM to acknowledge and be inclusive of a wide variety of population subgroups^2^.

Multiple studies have documented significant health disparities among SGM populations in high-income countries. Sexually transmitted infections (STIs), including human immunodeficiency virus (HIV)^3^, are more prevalent among SGM individuals – especially gay and bisexual men and transgender women, who likewise report lower use of preventive services that can potentially impair their wellbeing^4^. Studies have found that, when compared with heterosexual adults, sexual minority adults are more likely to report a number of health risk behaviors and health problems such as tobacco use^5^, obesity^6^, cardiovascular disease^7^, asthma^8^, and hyperglycemia^9^.

Gender minority individuals experience many of the same health disparities, with HIV/Aids being particularly prevalent among transgender women^10^. Previous work has shown that, compared to cisgender individuals, gender minorities are more likely to present with mood disorders (such as depression and anxiety), suicidal and nonsuicidal self-injury, physical inactivity, and tobacco use^11^. Studies have also indicated that gender minority adults, particular transgender women, may be at increased risk for cardiovascular disease compared to both cisgender men and women^12^.

SGM people are at higher risk of violence and victimization than their heterosexual counterparts^13^. A pervasive pattern of hate-motivated violence and sexual assault continues to be reported by SGM people worldwide^14^ despite an urgent call from the United Nations (UN) to end violence and discrimination against SGM people^15^. According to the World Health Organization (WHO), violence, abuse, discrimination, and social isolation are strongly associated with suicidal behavior, and global suicide rates are substantially higher among vulnerable groups such as SGM people^16^.

Studies addressing the health of SGM people in Chile are still scarce in the literature. A recent scoping review on the health of sexual minority women (SMW) in Latin America and the Caribbean^17^ found only two papers published in Chile. Although additional research has likely been published, these articles are not easy to find as they are often not indexed in the most common bibliographic databases – and thus not readily accessible to researchers and policy makers. Despite limited available literature, evidence indicates that Chilean SGM people may be at even higher risk for poor health outcomes when compared with other regions of the world. Such an assumption is based on the fact that homophobic and sexist attitudes appear to be more prominent in Latin America^18^. Gender ideology, or societal beliefs that legitimize gender inequality, has been much more widespread in the region and is believed to exacerbate such attitudes.

SGM people in Chile have been mostly invisible in health care settings and in public health policies, largely because sexual orientation and gender identity are viewed as binary. However, as a result of several social movements over the past two decades, authorities have implemented policies to promote respect for transgender and intersex people^19^. Despite increased visibility of SGM people in Latin American, health care models generally fail to recognize these population groups^20^. Heteronormativity and lack of knowledge about sexuality and gender on the part of health professionals is believed to be a primary reason for inadequate care provided to Chilean SGM people^21^. Gender ideology has been increasingly used worldwide to both describe and promote a movement against gender equality, the legal right to abortion, same-sex marriage and adoption, comprehensive sexuality education, and transgender rights. For instance, hours after taking office in January 2019, the Brazilian President Jair Bolsonaro removed SGM issues from the agenda of the Ministry of Human Rights – headed by Damares Alves, an ultraconservative evangelical pastor who claimed diversity policies have “threatened” the traditional Brazilian family^22^.

Since the creation of the human rights organization *Movimiento de Integración y Liberación Homosexual* (Homosexual Integration and Liberation Movement – MOVILH) in June 1991, a good deal of progress has been made in advancing the visibility and rights of SGM people in Chile^23^. In 2012, for example, the government passed a robust anti-discrimination bill that outlaws any discrimination that threatens the legitimate exercise of fundamental rights. Despite making no specific reference to sexual orientation or gender identity, such a bill was a major step in acknowledging SGM people as a social group worthy of protection. Chile has also allowed the civil union of same-sex couples since October 2015, providing these couples with many of the rights currently held by heterosexual couples. In December 2021 President Piñera signed legislation legalizing same-sex marriage. This law also permits same-sex couples to adopt children. In 2018, the Chilean Supreme Court issued the decision that transgender men and women could legally change their name and gender marker. In spite of these important social advances, SGM stigma and discrimination is still commonplace in Chile.

Studies on SGM health in Latin America are primarily focused on sexual health, particularly HIV/Aids^24,25^. Tat et al.^24^ conducted a systematic review on the sexual health of Brazilian SMW, verifying that increased risk for HIV and other STIs is primarily related to engaging in unsafe sexual activity with men. Research with sexual minority men (SMM) is also limited in the Latin American context; however, this group has received much more attention than SMW^26^. In a systematic review of 14 studies conducted by Albuquerque et al.^27^ to investigate barriers to healthcare access among SGM people, the authors found most of the studies were conducted in the US, with limited representation in Latin America. Similarly, a systematic review investigating suicide risk among SGM people identified 45 relevant studies, most of which were conducted in the US, Canada, the United Kingdom, and Australia, with only two studies in Latin America. The authors also verified a disproportional representation of SMM in the study samples^25^. Transgender people in Latin America are at increased risk of exposure to verbal, emotional, and physical violence, including hate crimes. A study conducted with transgender people in Latin America hypothesized that these negative experiences contribute to higher rates of anxiety, depression, suicidal ideation, HIV, and STIs among this group^28^. Other health risks among transgender adults are related to side effects of self-administered hormones, complications due to physical interventions and reproductive decisions made without adequate medical supervision and support^29^.

This study consists of a secondary data analysis of the most recent Chile National Health Survey^30^ – which was the first to include questions about sexual and gender identity – aiming to better understand the health concerns of SGM adults in Chile. We hypothesized that SGMs who participated in the survey would have poorer health outcomes than cisgender heterosexual participants. This study was reviewed and approved by the ethics committee of the *Pontificia Universidad Católica de Chile.*

## METHODS

### Study Population

Conducted between August 2016 and March 2017, the third Chile National Health Survey used a cross-sectional design and multistage stratified random sampling, resulting in a final sample of 6,233 participants aged 15 years or older, with national, regional, and urban/rural representativeness^30^. The main survey included 576 questions covering 40 health conditions, 35 risk and protective factors, and nine additional health problems. In addition, 25 biological measures were collected from 5,520 participants. Data were collected by means of four home visits, with the first, third, and fourth visits conducted by trained interviewers, whereas the second visit – when data on sexual orientation and gender identity was assessed for the first time – was conducted by a nurse^30^.

### Measures

**Sexual orientation:** was assessed by means of a single item measuring sexual attraction, which stated: “Sexual orientation is understood as the attraction that a person may have towards people of the opposite sex (heterosexual), same sex (homosexual), or towards both men and women (bisexual).” Then, participants were asked to select one of the following responses: (1) heterosexual (attraction towards people of the opposite sex); (2) gay/lesbian (attraction to people of the same sex); (3) bisexual (attraction to people of both sexes); (4) don't know; or (5) no answer^30^.

**Gender identity:** was explained through the following statement: “Gender identity refers to the way a person feels or identifies with respect to their gender.” Then, participants were asked to select one of the following responses: (1) male, (2) female, or (3) other. This question was combined with yet another addressing sex assigned at birth, whereby individuals whose gender identity did not match their sex assigned at birth were classified as transgender.

**Sexual health**: questions about sexual health included lifetime diagnosis of HIV and other STIs (e.g., gonorrhea, syphilis), HIV/STI testing in the past 12 months, number of sexual partners in lifetime, and age at first sexual intercourse.

**Depression:** depressive symptoms and depression prevalence were assessed using the Composite International Diagnostic Interview – Short Form (CIDI-SF), which covers the frequency of depressive symptoms experienced for at least two weeks in a row over the past 12 months, including dysphoria, anhedonia, fatigue, weight change, trouble sleeping, trouble concentrating, feelings worthlessness, and thoughts of death^31^. Scores were summed in a range of 0–8 using previously described methods, and participants reporting dysphoria or anhedonia with a CIDI-SF score greater than or equal to 5 met the criteria for probable depression (1 = “Probable depression”; 0 = “No depression”)^31^.

**Self-rated health:** was assessed using the 12-item Short-Form Health Survey (SF-12), a widely used measure of well-being and functional capacity of people over the age of 14. The SF-12 consists of eight domains, including limitations in physical activity because of health problems, bodily pain, general mental health, and vitality. The cumulative score (range 0–100) was obtained by summing scores across each domain, whereby higher scores represent better self-rated health.

### Statistical Analyses

All analyses were performed using Stata version 15.

**Descriptive analyses:** the overall study sample and each subgroup (i.e., heterosexual women, heterosexual men, lesbian women, gay men, bisexual women and men, questioning individuals, and transgender individuals) were described using frequencies, means, and standard deviations. The prevalence of probable depression, lifetime diagnosis of HIV and other STIs, and HIV/STI testing in the past 12 months were estimated within each subgroup, as well as the mean number of sexual partners in lifetime, age at first sexual intercourse, depressive symptoms, and self-rated health score.

**Regression analyses**: health outcomes of SGM and cisgender heterosexual participants were compared in a series of analyses. Differences in means of number of sexual partners, age at first sexual intercourse, number of depressive symptoms, and self-rated health scores among sexual and gender identity were assessed using age-adjusted linear regression models stratified by sex. Age-adjusted logistic regression models were used to examine sexual and gender identity differences in the odds of lifetime diagnosis of HIV and other STIs, HIV/STI testing in the past 12 months, and probable depression. Models comparing sexual minorities to cisgender heterosexual (reference group) adults were stratified by sex. Transgender adults were separately compared to cisgender heterosexual men and women. The frequencies, means, and standard deviations for all study variables are presented in Tables. The p-values from linear and logistic regressions were used to determine statistical significance across all analyses, whereby p-value less than 0.05 was considered significant.

## RESULTS

The number of participants decreased from 6,233 in the first visit (questionnaire 1) to 5,520 in the second visit (questionnaire 2)^26^ due to refusal to participate, inability to locate the participant, and no one being at home at the time of data collection. Upon refusal, a new interviewer was sent for a second and third attempt in hopes of gaining participation. Absolute sampling error was 2.6% nationwide and the root of the design effect was 1,797 – an estimate with 95% confidence and a relative error of less than 30%. The household response rate (number of complete interviews divided by the number of complete and partial interviews plus the number of non-interviews, such as refusals and unknown eligibility) was equal to 66% (67% of those eligible), and the participation rate was 90.2%^30^.

Female participants (62.9%) were more prevalent in the study sample in relation to male participants (37.1%). Regarding education, nearly one-fourth of participants (23.7%) had fewer than eight years of formal education, 53.3% had between 8 and 12 years, and 22% had 12 years or more. Among men, 96.7% identified themselves as heterosexual, 2.3% as gay, 0.7% as bisexual, 0.2% responded “I don't know,” and 0.1% did not answer the question on sexual orientation. Among women, 98.5% identified themselves as heterosexual, 0.6% as gay/lesbian, 0.2% as bisexual, 0.5% responded “I don't know,” and 0.2% did not answer. Only 0.3% of participants were transgender.

Table 1 summarizes results for men by sexual identity. Compared to heterosexual men, gay men were more likely to report having had HIV or other STIs in their lifetime (12.7% *versus* 1.1%,; p < 0.05) and to have been tested for HIV/STI in the past 12 months (45.3% *versus* 12.5%; p < 0.01). Men who did not respond to the question on sexual identity reported fewer sexual partners in their lifetime than heterosexual men (1.0 versus 1.2; p < 0.05). Moreover, gay men reported better self-rated health than heterosexual men (81.4 *versus* 64.6; p < 0.05). We found no statistically significant differences across study variables when comparing heterosexual to bisexual men, individuals who replied “I don't know” and those who did not respond.

**Table 1:** Comparison between sexual minority men and cisgender heterosexual men.

Sexual identity	Sexual health				Depression		Self-rated health
	Lifetime diagnosis of STIs % (95%CI)	Tested for HIV/STIs in the past 12 months % (95%CI)	Number of sexual partners in lifetime m (95%CI)	Age at first sexual intercourse m (95%CI)	Depressive symptoms m (95%CI)	Depression prevalence % (95%CI)	SF-12 score m (95%CI)
Cisgender heterosexual	1.1 (0.5 to 2.3)	12.5 (9.9 to 15.5)	1.2 (1.1 to 1.3)	16.6 (16.4 to 16.8)	0.7 (0.6 to 0.9)	10.2 (7.9 to 12.8)	64.6 (61.7 to 67.6)
Cisgender homosexual	12.7^a^ (0.5 to 49.3)	45.3^b^ (15.0 to 78.6)	2.5 (0.9 to 4.2)	15.7 (14.1 to 17.4)	0.7 (-0.4 to 1.8)	13.8 (0.7 to 50.9)	81.4 (68.0 to 94.9)
Cisgender bisexual	1.1 (0.0 to 6.3)	31.4 (6.5 to 68.6)	3.9 (0.9 to 6.8)	17.6 (14.1 to 21.0)	0.4 (-0.2 to 1.0)	7.6 (0.6 to 28.1)	61.7 (36.9 to 86.4)
I don't know	0	32.1 (1.1 to 87.5)	7.0 (-4.6 to 18.6)	17.5 (16.5 to 18.5)	0	0	73.0 (56.0 to 90.0)
No answer	0	22.2 (1.3 to 68.5)	1.0 (1.0 to 1.0)	16.7 (15.9 to 17.5)	0.7 (-0.3 to 1.7)	9.2 (0.2 to 43.0)	54.3 (32.4 to 76.2)

m: mean; 95%CI: 95% confidence interval; STIs: sexually transmitted infections; SF-12: 12-item short-form health survey (0-worst to 100-best).

a p < 0.05.

b p < 0.01.

Table 2 summarizes results for women by sexual identity. Bisexual women were less likely to have been tested for HIV/STIs in the past 12 months than heterosexual women (3.5% *versus* 21.4%; p < 0.01). Similar to men, women who did not respond to the sexual identity question reported and a lower mean of depressive symptoms in relation to heterosexual women (0.5 *versus* 1.5; p = 0.05). Although not statistically significant (p = 0.630), probable depression was more prevalent among bisexual women than heterosexual women (29.1% *versus* 21.6%).

**Table 2:** Comparison between sexual minority women and cisgender heterosexual women.

Sexual identity	Sexual health				Depression		Self-rated health
	Lifetime diagnosis of STIs % (95%CI)	Tested for HIV/STIs in the past 12 months % (95%CI)	Number of sexual partners in lifetime m (95%CI)	Age at first sexual intercourse m (95%CI)	Depressive symptoms m (95%CI)	Depression prevalence % (95%CI)	SF-12 score m (95%CI)
Cisgender heterosexual	1.2 (0.5 to 2.6)	21.4 (18.7 to 24.3)	0.9 (0.8 to 0.9)	18.5 (18.2 to 18.8)	1.5 (1.3 to 1.7)	21.6 (18.4 to 25.1)	59.4 (57.0 to 61.7)
Cisgender homosexual	-	14.4 (2.2 to 40.7)	0.9 (0.4 to 1.4)	17.2 (14.3 to 20.1)	1.7 (0.3 to 3.1)	13.8 (1.5 to 43.0)	52.5 (28.5 to 76.6)
Cisgender bisexual	-	3.5^a^ (0.5 to 11.4)	1.2 (0.8 to 1.5)	16.8 (15.4 to 18.2)	2.3 (0.8 to 3.7)	29.1 (4.7 to 68.8)	58.8 (35.2 to 82.3)
I don't know	-	-	0.7 (0.4 to 1.1)	20.8 (17.5 to 24.2)	1.4 (-0.2 to 3.0)	15.9 (0.9 to 55.5)	63.7 (50.4 to 77.0)
No answer	-	-	0.7 (0.2 to 1.3)	17.7 (13.0 to 22.3)	0.5 (-0.3 to 1.4)	5.6 (0.1 to 28.6)	54.8 (44.7 to 64.9)

m: mean; 95%CI: 95% confidence interval; STIs: sexually transmitted infections; SF-12: 12-item short-form health survey (0-worst to 100-best).

a p < 0.05.

Table 3 summarizes results for cisgender heterosexual and transgender participants. When compared with cisgender heterosexual men and women, transgender participants were more likely to meet criteria for probable depression, was only significant among men: transgender participants were at substantially greater risk than cisgender heterosexual (42.3% *versus* 10.2%; p = 0.01). Transgender participants reported worse self-rated health than both cisgender heterosexual.

**Table 3:** Comparison between transgender participants, cisgender heterosexual men, and cisgender heterosexual women.

Sexual identity	Sexual health				Depression		Self-rated health
	Lifetime diagnosis of STIs % (95%CI)	Tested for HIV/STIs in the past 12 months % (95%CI)	Number of sexual partners in lifetime m (95%CI)	Age at first sexual intercourse m (95%CI)	Depressive symptoms m (95%CI)	Depression prevalence % (95%CI)	SF-12 score m (95%CI)
Cisgender heterosexual men	1.1 (0.5 to 2.3)	12.5 (9.9 to 15.5)	1.2 (1.1 to 1.3)	16.6 (16.4 to 16.8)	0.7 (0.6 to 0.9)	10.2^b^ (7.9 to 12.9)	64.6^b^ (61.7 to 67.6)
Cisgender heterosexual women	1.2 (0.5 to 2.6)	21.5 (18.7 to 24.4)	0.9 (0.8 to 0.9)	18.5 (18.2 to 18.8)	1.5 (1.3 to 1.7)	21.5 (18.3 to 25.0)	59.5^a^ (57.1 to 61.8)
Transgender	0.6 (0.0 to 3.7)	8.8 (0.8 to 30.6)	1.6 (-0.4 to 3.5)	18.1 (16.9 to 19.4)	2.1 (0.4 to 3.9)	42.3 (10.4 to 80.1)	36.7 (18.1 to 55.3)

m: mean; 95%CI: 95% confidence interval; STIs: sexually transmitted infections; SF-12: 12-item short-form health survey (0-worst to 100-best).

a p < 0.05.

b p < 0.01.

## DISCUSSION

This is the first study to use nationally representative data to examine health disparities related to SGM health in Chile. Unfortunately, we were unable to corroborate differences in many comparisons with cisgender heterosexual men and women that have been described in the US and other high-income countries due to the small number of participants who self-identified as SGM.

Chile has made few attempts to estimate the size of its SGM population. According to 2019 World Bank, the country has around 19 million inhabitants^32^ – less than 2% of which identified themselves as being as lesbian, gay, or bisexual in a National Household Survey conducted by the Ministry of Social Development in 2015^33^. However, SGM organizations such as the MOVILH and *Fundación Iguales* believe this rate to be underestimated, because SGM people are often unwilling to identify themselves on national surveys. Of the 9,393 individuals between 15 and 29 years old who participated in the 8^th^ National Youth Survey published in 2015 by the National Institute of Youth of Chile, 82.9% identified themselves as heterosexual, 2.2% as homosexual, and 1.6% as bisexual, whereas 11.3% did not answer the question about sexual identity^33^. Studies conducted in the US have shown that participants who choose “I don't know,” “I prefer not to answer,” or those who refuse to answer questions about sexual orientation present significantly poorer health outcomes and higher behavioral risks than heterosexual participants^30^.

Despite the low representativeness of SGM participants within our study sample, we found evidence of health disparities in several important areas. Higher risk of HIV and other STIs among gay men and lower HIV/STIs testing among bisexual women were the most robust findings. Other studies conducted in Chile have found sexual minorities to present worse mental health and higher perceived discrimination rates^16^. In our study, transgender participants were more likely to meet the criteria for probable depression than cisgender heterosexual men, as well as to report lower self-rated health than cisgender heterosexual men and women.

Our findings indicate that gay men were more likely to be unemployed than heterosexual men, which is consistent with international data. According to the Williams Institute, one tenth of the SGM population in the US were unemployed in 2014–2017 and were more likely to live in poverty than cisgender heterosexual men^34^. This may be due to non-traditional gender roles and overall appearance, which makes some employers less likely to hire gay men. To better understand the employment conditions and socioeconomic status of Chilean SGM population, further studies with larger samples are required.

Corroborating studies conducted in other regions of the world, our results indicate that SGM people are more likely to report depression, which may be due primarily to the chronic stress associated with higher rates of stigma, discrimination, and victimization faced by these groups^35^.

Although limited, findings from the first national health survey to assess sexual orientation and gender identity in Chile suggest that Chilean SGM populations have poorer health than heterosexual cisgender individuals. However, we found fewer statistically significant differences than have been reported in most studies from other countries. As aforementioned, having a very small sample of SGM people and even smaller subsamples available for comparison likely obscured differences that would have been apparent in larger samples.

Aspects of data collection may have influenced the willingness of SGM participants to report their sexual and/or gender minority status. The means through which gender identity was assessed, for example, was particularly problematic: rather than including options for transgender man, woman, and non-binary, transgender participants were identified as those whose gender identity differs from their reported sex at birth. These factors indicate the persistent challenges in deepening knowledge about SGM health in Chile. Reliable estimates about the health of Chilean SGM people in relation to that of their cisgender, heterosexual counterparts will only be possible when more Chileans are willing to accurately report their SGM status in large-scale studies using probability samples, or upon the availability of methods to produce oversamples of SGM. In the meantime, studies using snowball or other social network-based sampling techniques and including specific questions about the lives and experiences of SGM people can provide valuable information on the topic. Information from both probability and non-probability samples are needed to better understand the health concerns of Chilean SGM people, as well as to support the development of guidelines and policies aimed at reducing health disparities related to sexual-orientation and gender-identity.
